# Comparison of tumor assessments using RECIST 1.1 and irRECIST, and association with overall survival

**DOI:** 10.1136/jitc-2021-003302

**Published:** 2022-02-28

**Authors:** Juliane Manitz, Sandra P D'Angelo, Andrea B Apolo, S Peter Eggleton, Marcis Bajars, Oliver Bohnsack, James L Gulley

**Affiliations:** 1 EMD Serono Research & Development Institute, Inc, Billerica, Massachusetts, USA, an affiliate of Merck KGaA; 2 Department of Medical Oncology, Memorial Sloan Kettering Cancer Center, New York, New York, USA; 3 Department of Medicine, Weill Cornell Medical College, New York, New York, USA; 4 Genitourinary Malignancies Branch, National Institutes of Health, Bethesda, Maryland, USA; 5 Merck Serono Ltd, Feltham, London, UK, an affiliate of Merck KGaA; 6 Merck Healthcare KGaA, Darmstadt, Germany; 7 Calyx, Berlin, Germany; 8 Genitourinary Malignancies Branch, Center for Cancer Research, National Cancer Institute, National Institutes of Health, Bethesda, Maryland, USA

**Keywords:** immunotherapy, clinical trials as topic

## Abstract

**Background:**

Patients treated with immune checkpoint inhibitors (ICIs) may experience pseudoprogression, which can be classified as progressive disease (PD) by Response Evaluation Criteria in Solid Tumors (RECIST) V.1.1 and could lead to inappropriate treatment discontinuation. Immune-response criteria were developed to better capture novel response patterns seen with ICIs.

**Methods:**

We pooled data from 1765 patients with 12 types of advanced solid tumors treated with avelumab (an anti-programmed death ligand 1 (PD-L1) monoclonal antibody) monotherapy in the JAVELIN Solid Tumor and JAVELIN Merkel 200 trials, conducted a comparative analysis of tumor assessments by investigators according to RECIST 1.1 and immune-related RECIST (irRECIST), and evaluated the correlation between progression-free survival (PFS) and overall survival (OS).

**Results:**

In total, 147 patients (8.3%) had a best overall response (BOR) of PD by RECIST 1.1 but had immune-related disease control by irRECIST (defined as immune-related BOR (irBOR) of immune-related stable disease or better). This discordance was seen irrespective of PD-L1 status and observed across all tumor types. Overall, PFS and immune-related PFS showed similar imputed rank correlations with OS.

**Conclusions:**

The use of irRECIST identified a subset of patients with a BOR of PD by RECIST 1.1 but an irBOR of immune-related disease control by irRECIST with a distinctive survival curve, thereby providing more clinically relevant information than RECIST 1.1 alone. However, as a surrogate endpoint for OS in the whole population, immune-related PFS by irRECIST did not show improved predictive value compared with PFS by RECIST 1.1.

## Background

Immune checkpoint inhibitors (ICIs) such as avelumab (anti-programmed death ligand 1 (PD-L1)) are an effective treatment option for various tumor types.^1 2^ ICIs activate the immune system, leading to unconventional response patterns.^1^ Response Evaluation Criteria in Solid Tumors (RECIST) V.1.1 guidelines are the gold standard for assessment of response, progression, or stability of disease experienced by patients with solid tumors resulting from anticancer treatment^3 4^; however, RECIST 1.1 does not capture unconventional response patterns, such as pseudoprogression, that are observed in a small percentage of patients who receive ICI treatment.^1^ Pseudoprogression is characterized by an initial increase in apparent tumor burden from baseline (which may be due to immune infiltrates, edema, and necrosis induced by ICI treatment), followed by a reduction in apparent tumor burden where the state of disease progression is not maintained at subsequent radiological assessment or confirmed by biopsy or clinical assessment.^1 3^ RECIST 1.1 considers pseudoprogression to be progressive disease (PD), potentially leading to inappropriate treatment discontinuation.^1 3^ Pseudoprogression has been reported in 2.8%–15.8% of patients in recent trials of anti-cytotoxic T-lymphocyte antigen 4 (CTLA4)/anti-PD-1 ICIs.^5–7^

Following reports of pseudoprogression with ICIs, immune-response criteria were developed.^3^ First, immune-related response criteria (irRC) were developed on the basis of WHO criteria, using bidimensional measurements.^8^ These were followed by immune-related RECIST (irRECIST)^9 10^ and immune RECIST,^11^ which were based on RECIST criteria using unidimensional measurements. Recently, assessments using immune-response criteria have been incorporated into several immunotherapy trials as primary or secondary endpoints.^12–16^ However, data to support whether immune-response criteria versus RECIST 1.1 better assess response to ICI treatment are limited to trials of single tumor types or relatively small sample sizes, or retrospective analyses.^16 17^ These data generally suggest that RECIST 1.1 and immune-response criteria do not provide substantially different assessments of response.^16^ However, some differences have been observed; in a recent, large retrospective analysis of patients treated with anti-PD-1/PD-L1 agents (N=4751), a small subgroup of patients who achieved PD by RECIST 1.1 achieved a complete or partial response (CR or PR) according to immune-response criteria (37 of 1693; 2.2%).^17^

Here, we report a comparative analysis of tumor assessments by RECIST 1.1 and immune-response criteria from patients with various advanced solid tumors who received avelumab monotherapy in the JAVELIN Solid Tumor and JAVELIN Merkel 200 trials.

## Methods

### Immune-related response criteria

When the first clinical studies of avelumab (the JAVELIN clinical program) were initiated, the only published immune-response criteria were irRC. The irRC criteria analyzed best overall response (BOR) by WHO criteria and immune-related BOR (irBOR) by irRC in patients with advanced melanoma who received ipilimumab (anti-CTLA4).^8^ Because we included multiple tumor types in our analysis, we modified the definition of irRC to include unidimensional measurements, allow consideration of non-target lesions, align assessment of lymph nodes with RECIST 1.1 techniques, and measure ≤5 target lesions at baseline as defined by RECIST 1.1.^4^ Subsequently, irRECIST criteria were published,^9 10^ and these are very similar to the immune-response criteria used in our analysis. Therefore, we refer to our criteria as irRECIST (online supplemental table 1).

10.1136/jitc-2021-003302.supp1 Supplementary data

The definitions of immune-related PD (irPD) by irRECIST (per the study protocols) and PD by RECIST 1.1 are shown in online supplemental table 2. The main differences include: for irRECIST, progression was not automatically defined by the appearance of a new lesion, and irPD was defined by an increase in the sum of the longest diameters of target and new measurable lesions by ≥20% (relative to the nadir, the smallest sum on study); for RECIST 1.1, PD can also be defined by an unequivocal increase in non-target lesions or ≥1 new lesion, and new measurable lesions are not included in the sum of the longest diameters.^4^ Furthermore, in this analysis, the definition of confirmation of irPD was amended *post hoc* to also consider discontinuation of imaging: irPD could also be confirmed by a second scan ≥4 weeks after the first irPD assessment, death, treatment discontinuation, initiation of follow-up treatment, or treatment reinitiation within 84 days after irPD assessment.

### Data set

This analysis pooled data from patients with histologically or cytologically proven metastatic or locally advanced solid tumors enrolled in the JAVELIN Solid Tumor trial (NCT01772004; data cut-off: February 15, 2017) and patients with histologically proven Merkel cell carcinoma (MCC) who had received ≥1 prior systemic therapy for metastatic MCC enrolled in the JAVELIN Merkel 200 trial (NCT02155647; data cut-off: March 24, 2017). Key eligibility criteria included patients who were ≥18 years old, were checkpoint inhibitor-naive, and had an Eastern Cooperative Oncology Group performance status (ECOG PS) of 0 or 1; full eligibility criteria for both trials have been published previously.^18 19^ Patients were enrolled irrespective of PD-L1 status (PD-L1 positivity was defined as PD-L1 expression in ≥1% of tumor cells using PD-L1 immunohistochemistry 73–10 pharmDx assay; Dako, Carpinteria, California). The castration-resistant prostate cancer cohort of the JAVELIN Solid Tumor study (n=18) was excluded from this analysis, as inclusion criteria for these patients did not mandate measurable disease at baseline.

All patients received avelumab monotherapy 10 mg/kg every 2 weeks until PD by RECIST 1.1, unacceptable toxicity, withdrawal, or other protocol-defined criteria for withdrawal (patients could continue treatment beyond PD, provided no significant clinical deterioration occurred); patients with a CR who had PD after stopping treatment could reinitiate avelumab treatment per investigator decision. Efficacy assessments included BOR, progression-free survival (PFS) by RECIST 1.1, irBOR and immune-related PFS (irPFS) by irRECIST, and overall survival (OS). Tumor assessments by both RECIST 1.1 and irRECIST were carried out by investigators every 6 weeks for 12 months, then every 12 weeks, to allow consideration for treatment decisions. Investigators could use their irRECIST assessments in those treatment continuation decisions.

### Identification of concordance/discordance between assessments by RECIST 1.1 and irRECIST

Assessments of BOR by RECIST 1.1 and irBOR by irRECIST were compared, and concordance between assessments by the two criteria was analyzed descriptively. Disease control was defined by RECIST 1.1 if BOR was CR/PR/stable disease (SD) and by irRECIST if irBOR was immune-related CR (irCR)/immune-related PR (irPR)/immune-related SD (irSD). Response was defined if BOR by RECIST 1.1 was CR/PR, and immune response was defined if irBOR was irCR/irPR. Initial PD was considered confirmed in the absence of further scans. The data were analyzed according to three subgroups defined by the presence or absence of a BOR/irBOR assessment of disease control: concordant disease control (agreement on BOR/irBOR assessments of disease control), concordant disease progression (agreement on BOR/irBOR assessments of progression), and discordant (BOR assessment of PD or not evaluable (NE) and irBOR assessment of disease control, ie, irSD or better). The subgroup definitions were based on an analysis by Wolchok *et al.* in which the association of OS with response was analyzed using WHO criteria and irRC in patients with ipilimumab-treated melanoma.^8^

The concordance and discordance between BOR and irBOR assessments according to the three subgroups was analyzed in the overall population and individual tumor types. OS according to the presence or absence of a BOR/irBOR assessment of disease control was also analyzed. Median OS and corresponding two-sided 95% CIs in each subgroup were calculated using the Kaplan-Meier method. To investigate the immortal time bias associated with the Kaplan-Meier analysis (whereby patients with a BOR of CR/PR or irBOR of irCR/irPR/irPD needed to be alive until the first tumor assessment and its confirmation), a 12-week landmark sensitivity analysis of OS was conducted. The landmark time point used was 89 days (allowing for two tumor assessments either to confirm response or irPD).

### Characterization of the discordant subgroup

The discordant subgroup was characterized in comparison to the concordant disease progression subgroup using descriptive statistical analysis of baseline characteristics. The cause of PD by RECIST 1.1 was also analyzed; frequencies of PD assessments in target, non-target, and new lesions were analyzed in the discordant subgroup in comparison to the concordant disease progression subgroup and the overall population.

### Association between irPFS/PFS and OS

Landmark OS according to the presence or absence of an early irPFS/PFS event (before day 89) was investigated. Additionally, rank correlations between OS and irPFS/PFS and corresponding two-sided 95% CIs were calculated for the overall population and for individual tumor types (online supplemental methods).

## Results

### Patients and treatment

A total of 1765 patients were included in this analysis (table 1); all patients had ≥3 months of follow-up, defined as the time from start of study treatment to analysis cut-off date. The data set comprised 12 solid tumor types, including adrenocortical carcinoma (ACC), colorectal cancer (CRC), gastric cancer/gastroesophageal junction cancer, MCC, melanoma, mesothelioma, metastatic breast cancer (MBC), non-small cell lung cancer (NSCLC), ovarian cancer (OC), renal cell carcinoma (RCC), squamous cell carcinoma of the head and neck, and urothelial carcinoma (UC) (online supplemental table 3). Overall, the median duration of avelumab treatment was 12.0 weeks (range, 2–173). After the first occurrence of PD by RECIST 1.1, 826 of 1765 patients (46.8%) had imaging, and 671 (38.0%) received subsequent anticancer treatment after RECIST PD (online supplemental table 4). In total, 550 patients (31.2%) had irPD during follow-up after having an irBOR of SD or better. Additionally, 31 patients had classical pseudoprogression, that is, any RECIST progression followed by irRECIST response at any later time point. In addition, 137 patients had atypical progression, that is, any RECIST progression followed by return to irSD or better.

**Table 1:** Baseline characteristics in the overall patient population and the discordant and concordant disease progression subgroups

	Discordant subgroup (n=147)	Concordant disease progression subgroup (n=798)	Overall (N=1765)
Median age (range), years	62.0 (23.0–89.0)	62.0 (21.0–91.0)	64.0 (19.0–91.0)
Sex, n (%)			
Female	70 (47.6)	403 (50.5)	852 (48.3)
Male	77 (52.4)	395 (49.5)	913 (51.7)
Race, n (%)			
White	111 (75.5)	612 (76.7)	1331 (75.4)
Other	36 (24.5)	184 (23.1)	430 (24.4)
Missing	0	2 (0.3)	4 (0.2)
Geographic region, n (%)			
America	98 (66.7)	564 (70.7)	1249 (70.8)
Asia	8 (5.4)	62 (7.8)	118 (6.7)
Europe	38 (25.9)	165 (20.7)	379 (21.5)
Missing	3 (2.0)	7 (0.9)	19 (1.1)
ECOG PS, n (%)			
0	61 (41.5)	256 (32.1)	671 (38.0)
1	86 (58.5)	542 (67.9)	1094 (62.0)
Tumor size at baseline, n (%)			
<Median	68 (46.3)	353 (44.2)	871 (49.3)
≥Median	79 (53.7)	441 (55.3)	886 (50.2)
Not available	0	4 (0.5)	8 (0.5)
Previous lines of therapy, n (%)			
0	59 (40.1)	250 (31.3)	691 (39.2)
1	30 (20.4)	232 (29.1)	435 (24.6)
>1	58 (39.5)	316 (39.6)	639 (36.2)
PD-L1 status, n (%)			
Negative	81 (55.1)	461 (57.8)	914 (51.8)
Positive	45 (30.6)	209 (26.2)	533 (30.2)
Not evaluable	21 (14.3)	128 (16.0)	318 (18.0)

ECOG PS, Eastern Cooperative Oncology Group performance status; PD-L1, programmed death ligand 1.

### Identification of concordance/discordance between assessments by RECIST 1.1 and irRECIST

Of the 1765 patients in this analysis, 147 (8.3%) made up the discordant subgroup and had a BOR of PD and an irBOR of disease control (table 2); 820 (46.5%) made up the concordant disease control subgroup and had both a BOR and irBOR of disease control; and 798 (45.2%) made up the concordant disease progression subgroup and had a BOR or irBOR of PD or irPD, respectively, or were NE. One case of PD/irCR was pseudoprogression, where disease progression was not confirmed at the next assessment; this patient initially had disease progression (PD by RECIST) and later had an irCR (table 2). The other six cases (three PR/irCR, one CR/irPR, and two SD/irCR) were due to data entry errors in the irRECIST assessments by the investigator (table 2). Of the remaining cases with variance, there were 8 cases of irPR with RECIST SD, 135 irSD with RECIST PD, and 11 irPR with RECIST SD. The reasons for this variance are not known but are likely due to expected differences between RECIST and irRECIST (eg, definition of PD per RECIST including new lesions or non-target lesion progression, meaning that patients had RECIST progression while PD criteria were not met based on irRECIST).

**Table 2:** Frequency (n) and proportion of the total population (%) of concordance/discordance between BOR assessed by RECIST 1.1 and irBOR assessed by irRECIST

irBOR by irRECIST, n (%)	BOR by RECIST 1.1, n (%)					
	CR	PR	SD	PD	NE	Overall
irCR	42 (2.4)	3 (0.2)†	2 (0.1)†	1 (<0.1)*	0	48 (2.7)
irPR	1 (<0.1)†	172 (9.7)	8 (0.5)	11 (0.6)	0	192 (10.9)
irSD	0	0	592 (33.5)	135 (7.6)	0	727 (41.2)
irPD	0	0	0	530 (30.0)	4 (0.2)	534 (30.3)
irNE	0	0	0	62 (3.5)	202 (11.4)	264 (15.0)
Overall	43 (2.4)	175 (9.9)	602 (34.1)	739 (41.9)	206 (11.7)	1765 (100)

The discordant subgroup (a BOR assessment of PD or NE and an irBOR assessment of disease control, ie, irSD or better) is shown: of patients with PD, 135 had irSD, 11 had irPR, and 1 had irCR (n=147).

*This patient had pseudoprogression; the patient initially had disease progression (PD by RECIST) and later had a CR (irCR).

†Due to data errors by the investigator in the assessment of BOR by RECIST 1.1.

BOR, best overall response; CR, complete response; irBOR, immune-related BOR; irCR, immune-related CR; irNE, immune-related NE; irPD, immune-related PD; irPR, immune-related PR; irRECIST, immune-related RECIST; irSD, immune-related SD; NE, not evaluable; PD, progressive disease; PR, partial response; RECIST, Response Evaluation Criteria in Solid Tumors; SD, stable disease.

**Figure 1:**
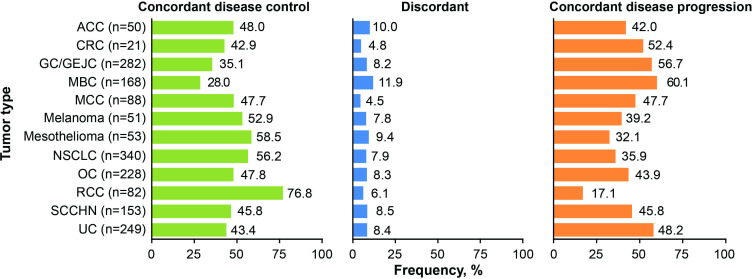
Indication-specific analysis of disease control by RECIST 1.1 and irRECIST. ACC, adrenocortical carcinoma; CRC, colorectal cancer; GC/GEJC, gastric cancer/gastroesophageal junction cancer; irRECIST; immune-related RECIST; MBC, metastatic breast cancer; MCC, Merkel cell carcinoma; NSCLC, non-small cell lung cancer; OC, ovarian cancer; RCC, renal cell carcinoma; RECIST 1.1, Response Evaluation Criteria in Solid Tumors V.1.1; SCCHN, squamous cell carcinoma of the head and neck; UC, urothelial carcinoma.

When considering only patients whose BOR was PD (n=739), most (n=530; 71.7%) had an irBOR of irPD, 62 (8.4%) were not evaluable by irRECIST, and 147 (19.9%) were classified as having an irBOR of disease control; however, most of these patients (n=135) had an irBOR of irSD so were not considered to be responders (or pseudoprogressors). Discordance in patients who had a BOR of PD and an irBOR of disease control was observed in all tumor types (figure 1). The frequency of discordance was relatively consistent across tumor types and ranged between 4.5% (MCC) and 11.9% (MBC).

**Figure 2:**
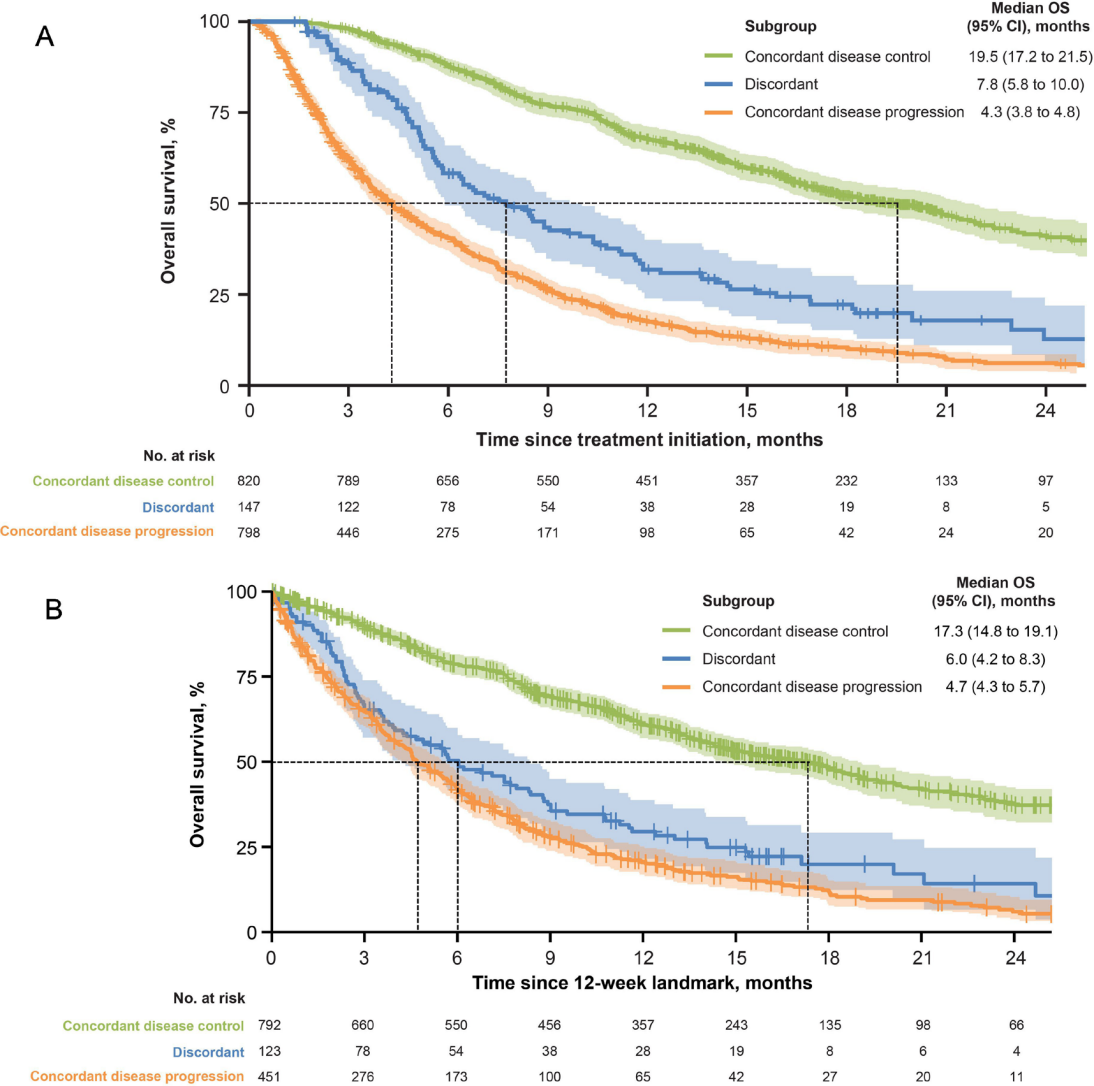
(A) Kaplan-Meier analysis of OS according to the presence or absence of a BOR or an irBOR assessment of disease control using RECIST 1.1 or irRECIST. (B) Twelve-week landmark sensitivity analysis. BOR, best overall response; irBOR, immune-related BOR; irRECIST; immune-related RECIST; OS, overall survival; RECIST 1.1, Response Evaluation Criteria in Solid Tumors V.1.1.

Kaplan-Meier estimates of OS according to the presence or absence of a BOR or irBOR assessment of disease control are shown in figure 2A. The survival curve for the discordant subgroup lay between the two concordant subgroups but closer to the concordant disease progression subgroup. Median OS (months) was as follows: discordant subgroup, 7.8 (95% CI, 5.8 to 10.0); concordant disease control subgroup, 19.5 (95% CI, 17.2 to 21.5), and concordant disease progression subgroup, 4.3 (95% CI, 3.8 to 4.8). Kaplan-Meier estimates of OS for the 12-week landmark sensitivity analysis are shown in figure 2B. Comparing the populations in the unadjusted Kaplan-Meier analysis in figure 2A and the 12-week landmark analysis in figure 2B, 399 patients were excluded. Of these, 316 OS events and 83 OS censorings occurred earlier than 89 days (12 weeks + 5-day window). In this sensitivity analysis, the survival curve for the discordant group was again situated between those of the concordant groups but closer to the disease progression group.

### Characterization of the discordant subgroup

Baseline characteristics and treatment and imaging between the discordant and concordant disease progression subgroups were well balanced (table 1 and online supplemental table 3); key laboratory values and biomarkers (serum levels of albumin, C-reactive protein, lymphocytes, neutrophils, platelets, and leukocytes; neutrophil/lymphocyte ratio; Fc-gamma receptor single-nucleotide polymorphisms; major histocompatibility complex class I and II genes; killer cell immunoglobulin-like receptor genes; and tumor mutational burden) were also balanced across subgroups (data not shown). In both subgroups, median patient age was 62.0 years, approximately 50% of patients were women, and approximately 75% were white. The proportion of patients with PD-L1–positive tumors was similar in the discordant and concordant disease progression subgroups (30.6% and 26.2%, respectively), indicating that discordance between BOR and irBOR assessments by RECIST 1.1 and irRECIST, respectively, was irrespective of tumor PD-L1 status. Some minor differences between the subgroups were observed; in the discordant versus concordant disease progression subgroup, a higher proportion of patients had an ECOG PS of 0 (41.5% vs 32.1%) and had received 0 lines of prior therapy (40.1% vs 31.3%).

Assessments of PD by RECIST 1.1 in target, non-target, and new lesions in the overall population and the discordant and concordant disease progression subgroups are shown in online supplemental figure 1. As expected, due to the different definition of PD per RECIST versus irRECIST, PD due to new lesions was more common in the discordant subgroup than in the concordant disease progression subgroup (58.5% vs 43.7%). PD due to target lesions was far less common in the discordant subgroup than in the concordant disease progression subgroup (7.5% vs 52.9%). PD due to non-target lesions (which was not accounted for by irPD by irRECIST) occurred in 25.0% of all patients, 28.9% of patients in the concordant disease progression subgroup, and 55.1% of patients in the discordant subgroup. Furthermore, although some patients had PD assessments due to only one type of lesion, others had multiple drivers for PD (target, non-target, and new lesions); overall, PD due to all criteria of RECIST 1.1 (target, non-target, and new lesions) was more common in the concordant disease progression subgroup (11.3%) than in the discordant subgroup (1.4%) and occurred in 5.8% of patients (n=103) overall.

### Association between irPFS/PFS and OS

**Figure 3:**
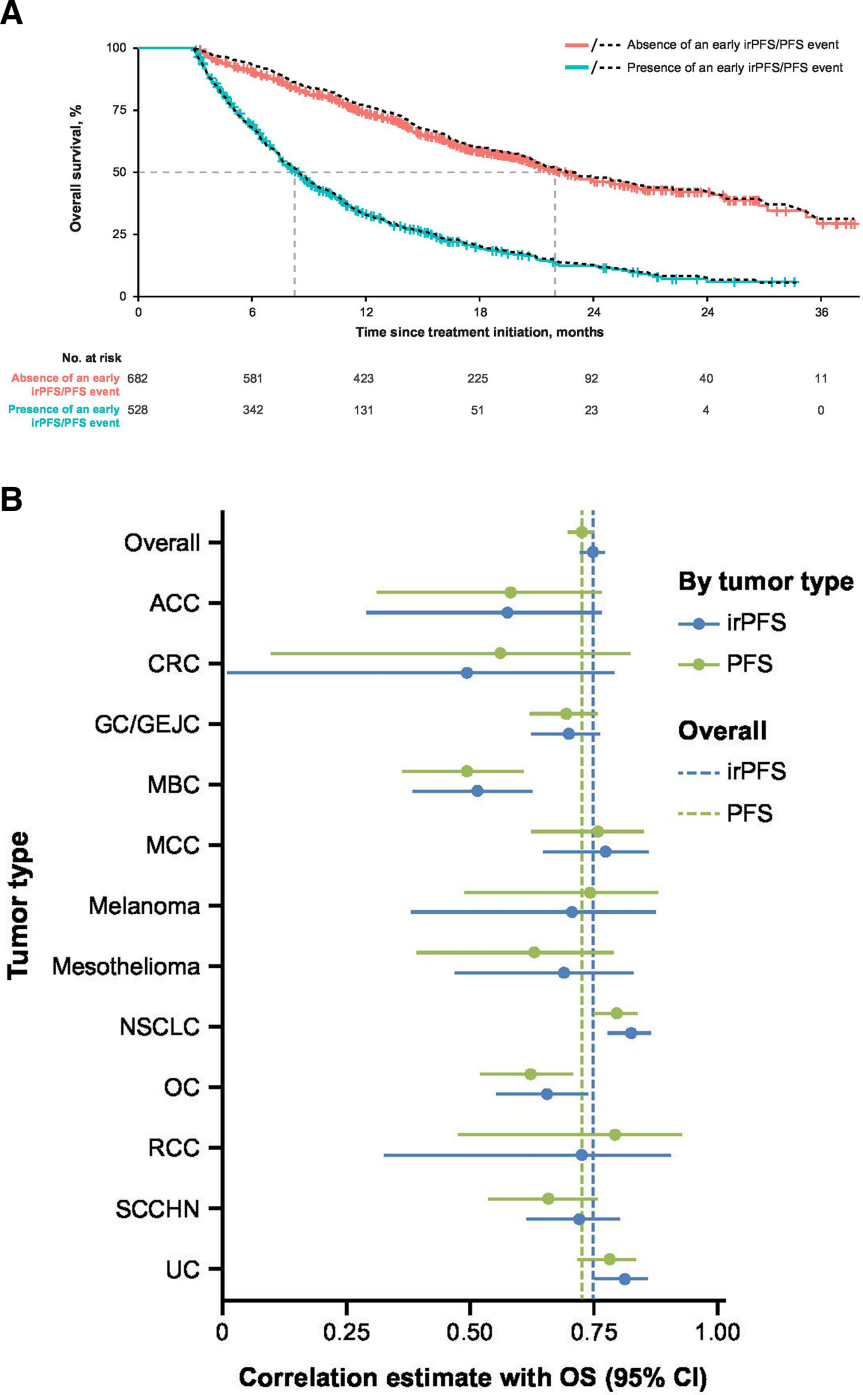
(A) Twelve-week landmark analysis of OS according to the presence or absence of an early irPFS/PFS event. Kaplan-Meier curves for landmark OS differentiating subgroups of patients with or without an early irPFS event are shown. An early irPFS event was defined as an irPFS event by irRECIST occurring before the 12-week landmark (before day 89). For reference, the dashed black lines refer to the respective subgroup definition based on early PFS events by RECIST 1.1 (before day 89). (B) Rank correlation analysis of PFS/irPFS and OS across tumor types. Spearman correlation coefficients for survival times under censoring, calculated using a semiparametric approach via copula-based estimation are shown. The null correlations for irPFS and PFS were 0.327 and 0.193, respectively (Pearson correlation, which assumes independent exponential distribution; for comparison only). ACC, adrenocortical carcinoma; CRC, colorectal cancer; GC/GEJC, gastric cancer/gastroesophageal junction cancer; irPFS, immune-related PFS; irRECIST, immune-related RECIST; MBC, metastatic breast cancer; MCC, Merkel cell carcinoma; NSCLC; non-small cell lung cancer; OC, ovarian cancer; OS, overall survival; PFS, progression-free survival; RCC, renal cell carcinoma; RECIST, Response Evaluation Criteria in Solid Tumors; SCCHN, squamous cell carcinoma of the head and neck; UC, urothelial carcinoma.

Kaplan-Meier estimates of OS according to the presence or absence of an early irPFS/PFS event based on a landmark analysis were conducted separately and are shown in figure 3A. Patients censored within 12 weeks were excluded from the analysis (110 patients for PFS by RECIST 1.1 and 210 for irPFS by irRECIST). The Kaplan-Meier estimates for OS in patients with early irPFS versus early PFS events were comparable, and the predictive value of PFS and irPFS for OS was similar. However, it must be noted that the validity of these results is subject to methodological limitations. The results were confirmed by an alternative approach that considered PFS events at any time point (figure 3B); in this analysis, the overall rank correlation estimate was similar for both PFS versus OS and irPFS versus OS: 0.727 (95% CI, 0.699 to 0.752) and 0.749 (95% CI, 0.723 to 0.773), respectively (null correlation, 0.193 and 0.327). The similar correlation was observed consistently across individual tumor types. An exception seems to be RCC; these data were immature with substantial censoring. The wide 95% CIs indicated small sample sizes and/or large variation within each tumor type. However, the observed correlation between PFS/irPFS and OS varies between the tumor types; for example, there were trends toward a lower correlation in the ACC, CRC, MBC, and OC tumors, and stronger correlations were observed in the NSCLC and UC tumors.

## Discussion

This analysis of a large number of patients with various tumor types confirmed reports of previous analyses that found the results of assessments by RECIST 1.1 and immune-response criteria are largely superimposable for most patients^1 16^; however, a discordant subgroup was also identified, which the authors feel should not be ignored. This subgroup included 8.3% of patients and comprised those who had a BOR of PD by RECIST 1.1 (or were NE) and an irBOR of disease control by irRECIST. Of note, most of these patients had BOR assessments of PD (or were NE) and irBOR assessments of irSD so were not considered responders and would not be denoted as pseudoprogressors. Discordance between BOR and irBOR assessments was observed in patients with every tumor type (ranging from 4.5% in MCC to 11.9% in MBC; figure 1) and regardless of PD-L1 status. In both the Kaplan-Meier analysis and 12-week landmark sensitivity analysis, the survival curve for the discordant subgroup lay between the two concordant subgroups, with median OS closer to that of the concordant disease progression subgroup; however, these analyses were impacted by immortal time bias. While the majority of the patients in the discordant subgroup did not have a response (irCR or irPR) per irRECIST, patients appeared to benefit from continued treatment. The type of response seen in these patients, which was an atypical response characterized by immune SD after RECIST PD, could therefore be used to identify patients who may benefit from continued treatment after RECIST progression.

Patients in the concordant disease progression subgroup had a worse performance status at baseline and were more likely to have received prior treatment than the discordant subgroup. However, on characterizing the discordant subgroup, no relevant differences in patient baseline characteristics could predict a discordance between BOR and irBOR assessments. We found that the discordant subgroup often had PD based on a single RECIST 1.1 criterion (non-target lesions; discordant group, 55.1%; concordant disease progression group, 28.9%). Furthermore, PD due to every criterion of RECIST 1.1 (target, non-target, and new lesions) was more common in the concordant disease progression subgroup (11.3%) than in the discordant subgroup (1.4%).

Considering the whole patient population, similar correlations between PFS and irPFS with OS were observed; therefore, the use of irRECIST may not have regulatory impact. However, these results may impact day-to-day clinical practice, particularly in the small subgroup in which differences exist. In these cases, irRECIST may offer additional guidance to physicians deciding whether to continue ICI treatment by identifying patients who are exhibiting a treatment benefit by irRECIST but not by RECIST 1.1. Continued monitoring of patients and consideration of other factors, such as clinical status, are critical to confirm non-PD and rule out hyperprogression (where the rate of progression is faster than the expected rate of tumor growth without ICI treatment).^1^

Previous publications have reported detailed evaluations of irRECIST, notably key manuscripts published by Nishino *et al.* in 2013 and 2014.^9 10^ However, both publications by Nishino included only patients with melanoma whereas our analysis included multiple tumor types. In the 2013 paper, the analysis compared unidimensional measurements versus bidimensional measurements, and in the 2014 paper, the analysis compared a maximum of 5 target lesions rather than 10^9 10^; consequently, a common approach in subsequent studies has been to amalgamate the methods of the 2013 and 2014 papers. Compared with this amalgamated approach, the only difference in our definition of irRECIST is the lack of requirement for confirmation of progression in our analyses (which was for operational rather than scientific reasons). We also note that the approach by Nishino *et al* did not consider non-target lesions, whereas in our analyses, unequivocal progression of non-target lesions, either alone or in combination with other features of immune progression, was classified as immune progression (irPD).

There were several limitations associated with our analyses. In the two clinical trials analyzed, response/PFS was primarily assessed by RECIST 1.1; assessments by irRECIST were conducted as secondary or exploratory endpoints. Furthermore, confirmation of progression by irRECIST required an additional scan, leading to more censoring observed in irPFS than PFS. Only 471 (26.7%) of all patients had imaging beyond an assessment of irPD by irRECIST; consequently, the definition of confirmation of irPD by irRECIST was amended *post hoc* to also consider discontinuation of imaging. An immortal time bias was associated with the Kaplan-Meier analysis of OS by BOR status; therefore, this analysis cannot be used to conclude whether using tumor assessments by RECIST 1.1 or irRECIST results in a stronger association with OS. To overcome the immortal time bias associated with the Kaplan-Meier analysis, a 12-week landmark analysis was conducted; however, a limitation of the 12-week landmark analysis was that patients with PFS/irPFS censoring before day 89 were excluded, and the number of early censoring differs for PFS by RECIST 1.1 versus irPFS by irRECIST. The analysis of the correlation between irPFS/PFS with OS was not subject to these limitations.

In conclusion, in this analysis, which combined data from 12 tumor types, we identified a discordant subgroup of patients who had a BOR of PD (or NE) by RECIST 1.1 and an irBOR of disease control by irRECIST, according to investigators. These results show important differences between assessments by RECIST 1.1 and irRECIST in a subgroup of patients that may be considered by physicians to better guide treatment decisions, where appropriate. They add to the growing body of evidence highlighting the need to use immune-response assessments for patients receiving ICIs. The authors call attention to the clinical implications of these data and recommend including time-sensitive irRECIST assessments in appropriate clinical trials and irPD to be considered by physicians as a criterion when deciding whether to discontinue ICI treatment.

## Data Availability

Data are available upon reasonable request. For all new products or new indications approved in both the European Union and the USA after January 1, 2014, Merck will share patient-level and study-level data after de-identification, as well as redacted study protocols and clinical study reports from clinical trials in patients. These data will be shared with qualified scientific and medical researchers, upon researcher’s request, as necessary for conducting legitimate research. Such requests must be submitted in writing to the company’s data sharing portal. More information can be found at https://www.merckgroup.com/en/research/our-approach-to-research-and-development/healthcare/clinical-trials/commitment-responsible-data-sharing.html. Where Merck has a co-research, co-development, or co-marketing/co-promotion agreement or where the product has been out-licensed, it is recognized that the responsibility for disclosure may be dependent on the agreement between parties. Under these circumstances, Merck will endeavor to gain agreement to share data in response to requests.
