# Supplementary material for: Comparison of tumor assessments using RECIST 1.1 and irRECIST, and association with overall survival
Source: J Immunother Cancer. 2022 Feb 28;10(2):e003302. doi: 10.1136/jitc-2021-003302 (PMC8886415; doi:10.1136/jitc-2021-003302)
Supplement: Supplementary data [file jitc-2021-003302supp001.pdf]

Supplement

Supplementary Methods

- The rank correlations between OS and PFS by RECIST 1.1, as well as OS and irPFS by irRECIST, were derived using a bivariate semiparametric model that takes into account censoring of the variables[1]
- First, censored observations in PFS, irPFS, and OS were augmented using Gaussian copulas, and then the Spearman rank correlation coefficient for the imputed variables was calculated using an iterative multiple imputation method provided by Schemper et al [1]
- For comparison, respective null Pearson correlations were calculated under the assumption of independent exponential distributions,[2] which can be derived as the fraction between median PFS and median OS:
  - $\rho_0(PFS, OS) = \frac{median(PFS)}{median(OS)}$
- Of note, the Pearson correlation is generally lower than the Spearman rank correlation

Supplementary References

1. Schemper M, Kaider A, Wakounig S, et al. Estimating the correlation of bivariate failure times under censoring. *Stat Med* 2013;32:4781-4790.
2. Fleischer F, Gaschler-Markefski B, Bluhmki E. A statistical model for the dependence between progression-free survival and overall survival. *Stat Med* 2009;28:2669-2686.
3. Eisenhauer EA, Therasse P, Bogaerts J, et al. New response evaluation criteria in solid tumours: revised RECIST guideline (version 1.1). *Eur J Cancer* 2009;45:228-247.
4. Nishino M, et al. *Clin Cancer Res* 2013;19:3936-3943.

Manitz J, *et al. J Immunother Cancer* 2022; 10:e003302. doi: 10.1136/jitc-2021-003302

Supplementary Table 1. Comparison of RECIST 1.1 and irRECIST

RECIST 1.1[3]	irRECIST*
CR	irCR
• Complete disappearance of all non-nodal lesions and return of nodal lesions to normal size without new lesions<br>• Pathological lymph nodes (target or nontarget) must have reduction in short axis to <10 mm<br>• Confirmed by second scan ≥4 weeks after CR assessment†	• Complete disappearance of non-nodal lesions and return of nodal lesions to normal size without new lesions<br>• Measurable lymph nodes must have reduction in short axis to ≤10 mm<br>• Confirmed by second scan ≥4 weeks after irCR assessment
PR	irPR
• Decrease in sum of longest diameters of target lesions by ≥30% (compared with baseline)<br>• No marked increase in nontarget lesions or no new lesions<br>• Confirmed by second scan ≥4 weeks after PR assessment†	• Decrease in sum of longest diameters of target and new measurable lesions by ≥30% (compared with baseline)<br>• No marked increase in nontarget lesions<br>• Confirmed by second scan ≥4 weeks after irPR assessment
SD	irSD
• Neither CR/PR nor PD	• Neither irCR/irPR nor irPD
PD	irPD

• Increase in the sum of longest diameters of target lesions by ≥20% relative to smallest sum on study (including baseline)<br>• Absolute increase in sum of longest diameters of ≥5 mm<br>• Unequivocal increase in nontarget lesions<br>• Appearance of ≥1 new lesion(s)	• Increase in the sum of longest diameters of target and new measurable lesions by ≥20% (compared with nadir)<br>• Absolute increase in sum of longest diameters of ≥5 mm<br>• Confirmed by second scan ≥4 weeks after irPD assessment; death, treatment discontinuation, initiation of follow-up treatment, or treatment reinitiation within 84 days after irPD assessment; or discontinuation of imaging

CR, complete response; irCR, immune-related CR; irPD, immune-related PD; irPR, immune-related PR; irRECIST, immune-related Response Evaluation Criteria in Solid Tumors; irSD, immune-related SD; PD, progressive disease; PR, partial response; SD, stable disease.

* irRECIST criteria used in this analysis were defined according to the study protocols and were similar to the definition of irRECIST by Nishino M, et al [4]

† Required for confirmed CR/PR; frequency of tumor reevaluation is specified by the trial protocol.

Supplementary Table 2. Comparison of disease progression assessed by RECIST 1.1 and irRECIST

RECIST 1.1[3]	irRECIST*
PD	irPD
• Increase in the sum of longest diameters of target lesions by ≥20%, relative to smallest sum on study (including baseline)<br>• Absolute increase in sum of longest diameters of ≥5 mm<br>• Unequivocal increase in nontarget lesions<br>• Appearance of ≥1 new lesion(s)	• Increase in the sum of longest diameters of target and new measurable lesions by ≥20% (compared with nadir)<br>• Absolute increase in sum of longest diameters of ≥5 mm<br>• Confirmed by second scan ≥4 weeks after irPD assessment; death, treatment discontinuation, initiation of follow-up treatment, or treatment reinitiation within 84 days after irPD assessment; or discontinuation of imaging

irRECIST; immune-related RECIST; irPD, immune-related PD; PD, progressive disease; RECIST 1.1, Response Evaluation Criteria in Solid Tumors version 1.1

* irRECIST criteria used in this analysis were defined according to the study protocols and were similar to the definition of irRECIST by Nishino M, et al.[4]

Supplementary Table 3. Tumor types in the concordant disease control, discordant, and concordant disease progression subgroups

Patients, n (%)	ACC (n=50)	CRC (n=21)	GC/GEJC (n=282)	MBC (n=168)	MCC (n=88)	Melanoma (n=51)	Mesothelioma (n=53)	NSCLC (n=340)	OC (n=228)	RCC (n=82)	SCCHN (n=153)	UC (n=249)	Overall (N=1765)
Concordant disease control subgroup	24 (48.0)	9 (42.9)	99 (35.1)	47 (28.0)	42 (47.7)	27 (52.9)	31 (58.5)	191 (56.2)	109 (47.8)	63 (76.8)	70 (45.8)	108 (43.4)	820 (46.5)
Discordant subgroup	5 (10.0)	1 (4.8)	23 (8.2)	20 (11.9)	4 (4.5)	4 (7.8)	5 (9.4)	27 (7.9)	19 (8.3)	5 (6.1)	13 (8.5)	21 (8.4)	147 (8.3)
Concordant disease progression subgroup	21 (42.0)	11 (52.4)	160 (56.7)	101 (60.1)	42 (47.7)	20 (39.2)	17 (32.1)	122 (35.9)	100 (43.9)	14 (17.1)	70 (45.8)	120 (48.2)	789 (45.2)

ACC, adrenocortical carcinoma; CRC, colorectal cancer; GC/GEJC, gastric cancer/gastroesophageal junction cancer; MBC, metastatic breast cancer; MCC, Merkel cell carcinoma; NSCLC; non-small cell lung cancer; OC, ovarian cancer; RCC, renal cell carcinoma; SCCHN, squamous cell carcinoma of the head and neck; UC, urothelial carcinoma.

Manitz J, *et al. J Immunother Cancer* 2022; 10:e003302. doi: 10.1136/jitc-2021-003302

Supplementary Table 4. Treatment and imaging in the overall patient population and in the discordant and concordant disease progression subgroups

	Discordant subgroup (n=147)	Concordant disease progression subgroup (n=798)	Overall (N=1765)
Median DOT (range), weeks	12.0 (2.0-97.9)	6.00 (2.00-124)	12.0 (2.00-173)
Treatment, n (%)			
Beyond PD			
Yes	106 (72.1)	303 (38.0)	671 (38.0)
No	41 (27.9)	495 (62.0)	1094 (62.0)
Beyond irPD			
Yes	32 (21.8)	289 (36.2)	541 (30.7)
No	115 (78.2)	509 (63.8)	1224 (69.3)
Imaging timepoints, n (%)			
Beyond PD			
Yes	108 (73.5)	400 (50.1)	826 (46.8)
No	39 (26.5)	199 (24.9)	417 (23.6)
Missing	0	199 (24.9)	522 (29.6)
Beyond irPD			
Yes	28 (19.0)	247 (31.0)	471 (26.7)
No	43 (29.3)	295 (37.0)	545 (30.9)
Missing	76 (51.7)	256 (32.1)	749 (42.4)
Tumor shrinkage by ≥5%, n (%)			
After PD			

Yes	54 (36.7)	206 (25.8)	400 (22.7)
No	93 (63.3)	592 (74.2)	1365 (77.3)
After irPD			
Yes	22 (15.0)	231 (28.9)	398 (22.5)
No	125 (85.0)	567 (71.1)	1367 (77.5)

DOT, duration of treatment; irPD, immune-related PD; PD, progressive disease.

Supplementary Figure 1. PD assessed by RECIST 1.1 in target, nontarget, and new lesions in the overall population and in the discordant and concordant disease progression subgroups

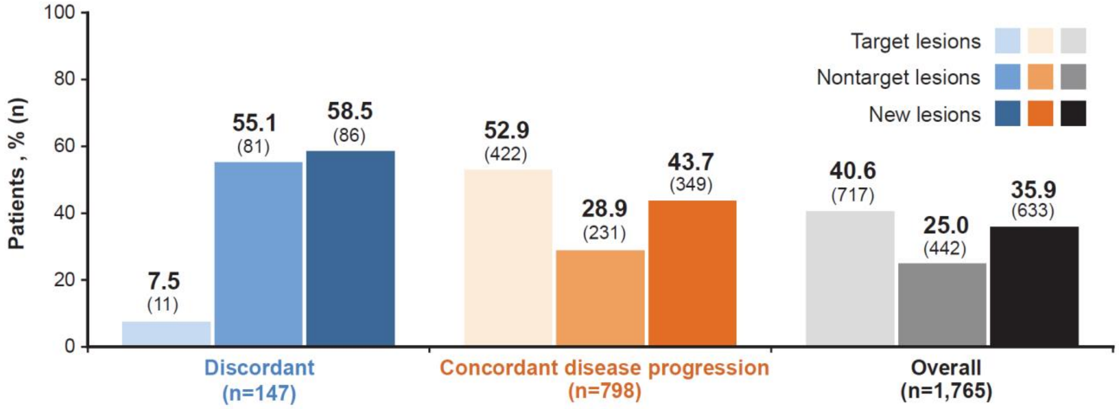

PD, progressive disease; RECIST 1.1, Response Evaluation Criteria in Solid Tumors version 1.1.

Manitz J, *et al. J Immunother Cancer* 2022; 10:e003302. doi: 10.1136/jitc-2021-003302
